# Phylogenetic and full-length genome mutation analysis of SARS-CoV-2 in Indonesia prior to COVID-19 vaccination program in 2021

**DOI:** 10.1186/s42269-021-00657-0

**Published:** 2021-11-21

**Authors:** Reviany V. Nidom, Setyarina Indrasari, Irine Normalina, Astria N. Nidom, Balqis Afifah, Lestari Dewi, Andra K. Putra, Arif N. M. Ansori, Muhammad K. J. Kusala, Mohammad Y. Alamudi, Chairul A. Nidom

**Affiliations:** 1 Coronavirus and Vaccine Formulation Research Group, Professor Nidom Foundation, Surabaya, Indonesia; 2 Riset AIRC Indonesia, Surabaya, Indonesia; 3 Faculty of Medicine, Universitas Hang Tuah, Surabaya, Indonesia; 4 Dr. Ramelan Naval Hospital, Surabaya, Indonesia; 5 Program Pendidikan Magister Menuju Doktor Untuk Sarjana Unggul (PMDSU) Program - Batch III, Ministry of Education, Culture, Research, and Technology, Jakarta, Indonesia; 6 Faculty of Veterinary Medicine, Universitas Airlangga, Surabaya, Indonesia

**Keywords:** COVID-19, Genome analysis, Indonesia, SARS-CoV-2, Spike protein

## Abstract

**Background:**

Indonesia has started the big project of COVID-19 vaccination program since 13 January 2021 by employing the first shot of vaccine to the President of Indonesia as the outbreak and rapid transmission of COVID-19 have endangered not only Indonesian but the global health and economy. This study aimed to investigate the full-length genome mutation analysis of 166 Indonesian SARS-CoV-2 isolates as of 12 January 2021.

**Results:**

All data of the isolates were extracted from the Global Initiative on Sharing All Influenza Data (GISAID) EpiCoV database. CoVsurver platform was employed to investigate the full-length genome mutation analysis of all isolates. This study also focused on the phylogeny analysis in unlocking the mutation of S protein in Indonesian SARS-CoV-2 isolates. WIV04 isolate that was originated from Wuhan, China was used as the virus reference according to the CoVsurver default. The result showed that a full-length genome mutation analysis of 166 Indonesian SARS-CoV-2 isolates was successfully generated. Every single mutation in S protein was described and then visualized by utilizing BioRender platform. Furthermore, it also found that D614G mutation appeared in 103 Indonesian SARS-CoV-2 isolates.

**Conclusions:**

To sum up, this study helped to observe the spread of COVID-19 transmission. However, it also proposed that the epidemiological surveillance and genomics studies might be improved on COVID-19 pandemic in Indonesia.

**Supplementary Information:**

The online version contains supplementary material available at 10.1186/s42269-021-00657-0.

## Background

SARS-CoV-2 firstly occurred in China and then transmitted sporadically worldwide. In March 2020, WHO announced that its infection was a pandemic. COVID-19 outbreak and rapid transmission have endangered global health and economy (Khan et al. 2020a, b; Wu et al. 2020) including Indonesia (Gunadi et al. 2020, 2021). This crisis has called for an extensive scientific mobilization of researches on SARS-CoV-2 focusing on its clinical aspects, its characteristics, and its mechanism of transmission, with the ultimate aim of counteracting the devastating outcomes (Huang et al. 2020; Li et al. 2020). Previously, there were six coronaviruses that infected humans, these are HCoV-229E (1966), HCoV-OC43 (1967), SARS-CoV (2002), HCoV-NL63 (2004), HCoV-HKU1 (2005), and MERS-CoV (2012) (Artika et al. 2020). Recently, around 196 million people globally have been infected by the seventh coronavirus called SARS-CoV-2 (COVID-19), with more than 4 million deaths as a result of this pandemic. In Indonesia, there are more than 3.3 million cases and around 92,000 people died. These data were derived from CSSE at Johns Hopkins University online website as 30 July 2021 which tracks COVID-19 cases in real-time (Dong et al. 2020).

As for the coronaviruses themselves, the family *Coronaviridae* is categorised into four different genera: *Gammacoronavirus*, *Deltacoronavirus*, *Betacoronavirus*, and *Alphacoronavirus*. Both animals and humans can be infected by coronaviruses (Ou et al. 2020). SARS-CoV-2 genome is a single-stranded positive-sense RNA of roughly 30,000 nucleotides with four structural proteins encoded by the genome and spike (S) protein is the most important one (Shereen et al. 2020). It is because S protein is the primary target antigen in the SARS-CoV-2 vaccine (Phan 2020). Previously, our study revealed the candidate for a peptide-based vaccine against the virus was identified based on the four structural proteins (Ansori et al. 2020; Normalina et al. 2020). Thus, it is very important to investigate the S protein from Indonesian SARS-CoV-2 isolates.

Many significant variants of SARS-CoV-2 appeared in the end of 2020 (Ansori et al. 2021). D614G mutation ever became the spotlight in the early time of COVID-19 pandemic and had a high correlation with the widespread infection and virulence besides the changes of antigenicity (Korber et al. 2020; Nidom et al. 2020a, b). Mutations on SARS-CoV-2 need to be highly supervised and mapped with the sole goal in overcoming COVID-19 pandemic (Tegally et al. 2021). These days WHO had mapped four variants which needed to be attention, there were the variants of Alpha, Beta, Gamma, and Delta (Duong 2021). Delta variant has caused an increasing crisis in Indonesia after hitting India for about two months (May 2021). The number of cases in Indonesia has sharply arisen over the last month (July 2021) and kept rising (Dyer 2021; Kupferschmidt and Wadman 2021).

Recently, many attempts have been made by scientists to generate vaccines to fight against SARS-CoV-2 worldwide with protein-based vaccines as the most advanced types and the private sector is at the forefront of these studies (Belete 2020; Callaway 2020; Nidom et al. 2020b; van Riel and de Wit 2020). However, the mutation rate of *Coronaviridae* is reminding high as various studies reported the mutation might implicate the efficacy of vaccines or other therapeutic strategies. Mutations on variants or lineages have appeared in several countries around the world which need to be mapped (Chen et al. 2021; Greaney et al. 2021; Weisblum et al. 2020) including in Indonesia. Thus, investigating the phylogenetic and full-length genome mutation analysis of 166 Indonesian SARS-CoV-2 isolates became the goal of this study.

## Methods

### SARS-CoV-2 isolates

The data extraction of all Indonesian SARS-CoV-2 isolates (166 isolates) from Global Initiative on Sharing All Influenza Data (GISAID) EpiCoV database (https://www.gisaid.org/) was completed on 12 January 2021. This study only used the complete genome and high coverage criteria according to the GISAID EpiCoV standard. All of 166 Indonesian SARS-CoV-2 isolates were derived from various provinces in Indonesia, for example: Special Region of Aceh, North Sumatra, Lampung, Banten, Special Capital Region of Jakarta, West Java, Central Java, Special Region of Yogyakarta, East Java, and so on (Additional file 1). This study identified the total isolates in every province, GISAID clades, and lineages. All data were visualized using GraphPad Prism software v.9.2 (GraphPad Software, Inc., California, USA). Additionally, WIV04 isolate (GISAID clade: L; lineage: B) was collected from a female retailer at Huanan Seafood Wholesale Market, submitted by the Wuhan Institute of Virology, Chinese Academy of Sciences in China and applied as a virus reference based on CoVsurver default in this study (Sengupta et al. 2021).

### Full-length genome mutation analysis

Every single gene of 166 Indonesian SARS-CoV-2 isolates was investigated, such as NSP1-16, S, NS3, E, M, NS6, NS7a, NS7b, NS8, and N. In addition, the application of CoVsurver platform (https://www.gisaid.org/epiflu-applications/covsurver-mutations-app) was applied to investigate the full-length genome mutation analysis of all isolates (Sengupta et al. 2021). Besides, the mapping of D614G mutation from the isolates derived from the database was also performed in this study based of the study from Korber et al. (2020). All data were visualized using GraphPad Prism software v.9.2 (GraphPad Software, Inc., California, USA) and BioRender platform (https://www.biorender.com).

### 3D structure visualization

In this study, 3D structure visualization of SARS-CoV-2 S protein was rendered by utilizing SWISS-MODEL web server (https://swissmodel.expasy.org) and PyMOL v2.4 (Schrödinger, Inc, New York, USA) with professional license for academic (Gurung 2020; Raj 2020). Then, the schematic diagram was edited with BioRender platform (https://www.biorender.com) (Ansori et al. 2020). This method was employed to easily identify the location of various mutations in SARS-CoV-2 S protein.

### Molecular phylogenetic analysis

The molecular phylogenetic modelling and tree visualization was rendered by applying MEGA X software (Pennsylvania State University, USA) to the maximum likelihood method using the Indonesian and other coronavirus isolates from other places such as Nepal, Sri Lanka, Russia, Nigeria, Serbia, Bangladesh, Greece, Poland, Morocco, Czech Republic, the Netherland, Germany, Saudi Arabia, Egypt, South Africa, Malaysia, Guam, France, Australia, Puerto Rico, Tunisia, Chile, Vietnam, United States, Uruguay, Thailand, Timor-Leste, Spain, Kenya, Japan, Pakistan, Kazakhstan, Colombia, Jamaica, Hong Kong, Israel, Italy, Brazil, China, Turkey, South Korea, India, Sweden, and Iran. Furthermore, coronaviruses derived from bat, pangolin, mink, and other coronaviruses isolated which had infected human previously were also utilized as the comparison. The molecular phylogenetic was tested by 1000 bootstrapped input datasets and cross-referencing it with the Tamura-Nei substitution model (Ansori et al. 2021; Normalina et al. 2020; Sohpal 2020; Stefanelli et al. 2020).

## Results

### Full-length genome mutation analysis and 3D structure visualization

**Fig. 1:**
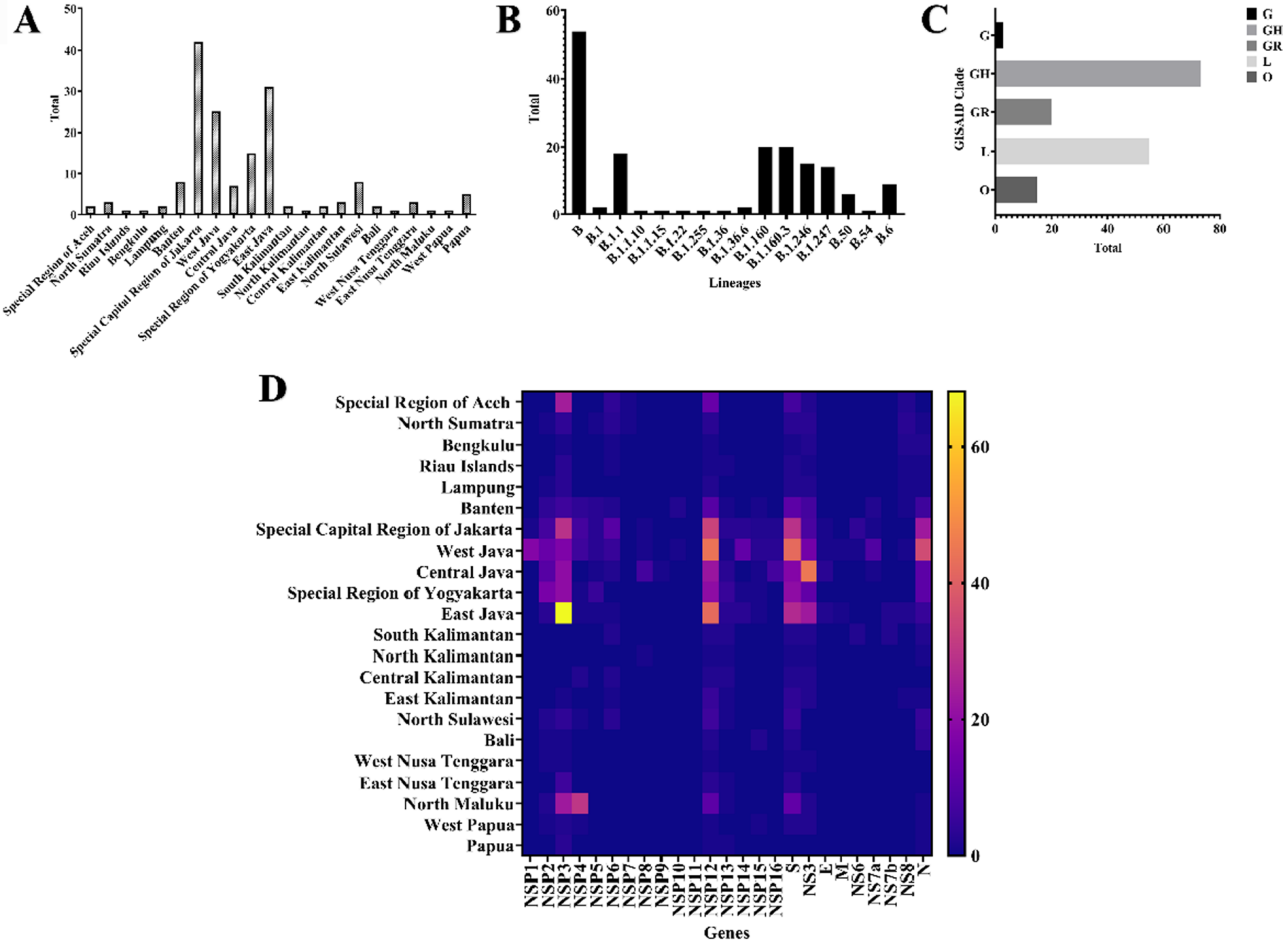
All Indonesian SARS-CoV-2 isolates data. **A** The origin of isolates; **B** the lineages distribution of the isolates; **C** the GISAID EpiCoV clades; and **D** the full-length genome mutation analysis of all isolates used in this study. All data were visualized using GraphPad Prism software

This study took 166 SARS-CoV-2 isolates from various regional areas in Indonesia, such as Special Region of Aceh (*n* = 2), North Sumatra (*n* = 3), Riau Islands (*n* = 1), Bengkulu (*n* = 1), Lampung (*n* = 2), Banten (*n* = 8), Special Capital Region of Jakarta (*n* = 42), West Java (*n* = 25), Central Java (*n* = 7), Special Region of Yogyakarta (*n* = 15), East Java (*n* = 31), South Kalimantan (*n* = 2), North Kalimantan (*n* = 1), Central Kalimantan (*n* = 2), East Kalimantan (*n* = 3), North Sulawesi (*n* = 8), Bali (*n* = 2), West Nusa Tenggara (*n* = 1), East Nusa Tenggara (*n* = 3), North Papua (*n* = 1), West Papua (*n* = 1), and Papua (*n* = 5) (Fig. 1A and Additional file 1). Various lineages of SARS-CoV-2 are also mapped in this study. As a result, this study identified 14 lineages from 166 isolates and the Lineage B (*n* = 54) was dominant in Indonesia (Fig. 1B). Significantly, the isolates were grouped in the G (*n* = 3), GH (*n* = 73), GR (*n* = 20), L (*n* = 55), and O (*n* = 15) clades. This result also revealed those lineages of SARS-CoV-2 isolates in Indonesia according to the accumulated data from GISAID EpiCoV database and found that Clade GH was dominant in Indonesia (Fig. 1C).

This study also analyzed the full-length genome mutation analysis of all isolates using a heat map data (Fig. 1D and Additional file 1). Based on the data, non-structural protein 3 (NSP3) became the most frequent mutation compared to all genes in 166 Indonesian SARS-CoV-2 isolates. In line with this, various genes, such as NSP12, NS3, and S protein, also were found to have high mutation numbers.

**Fig. 2:**
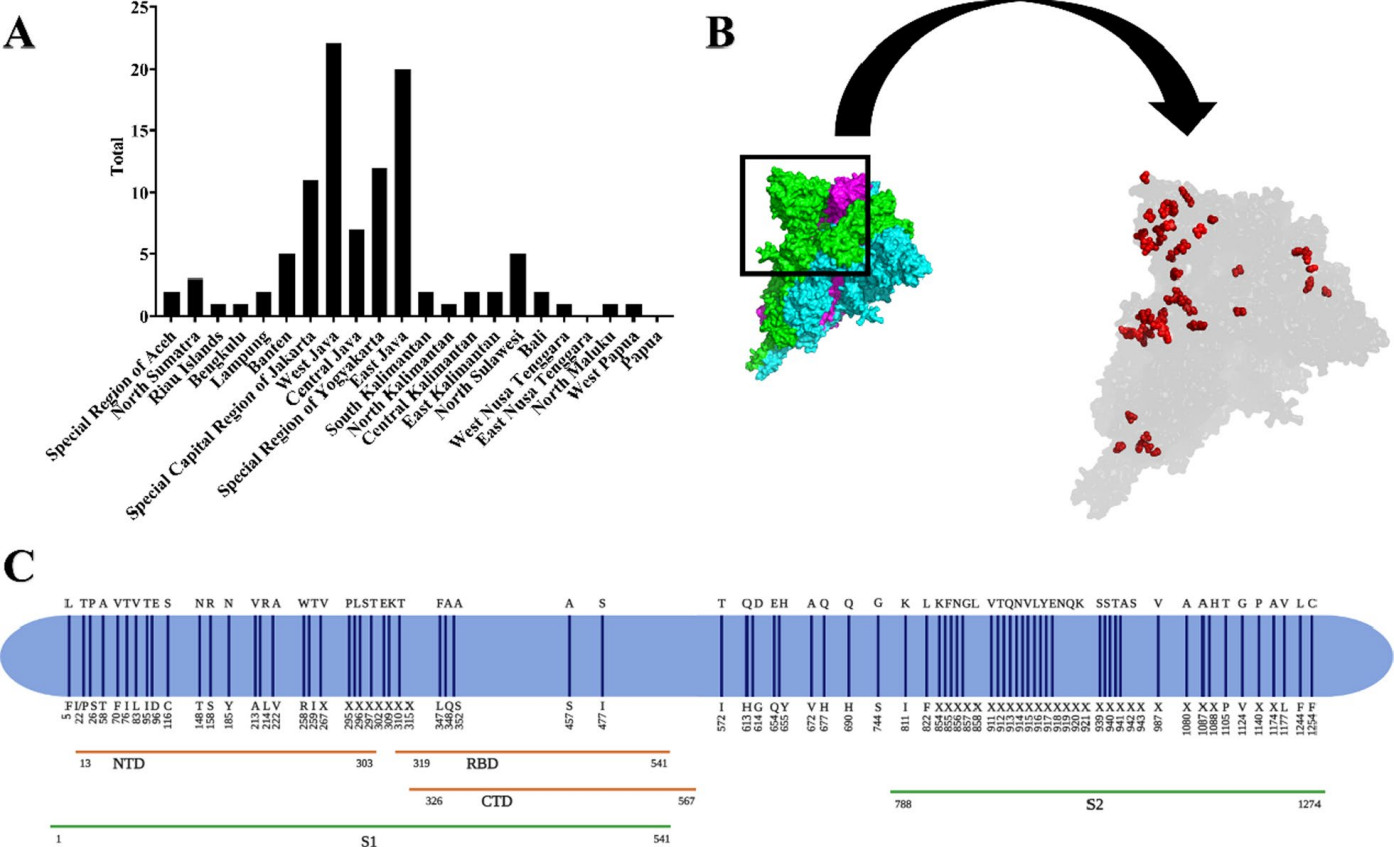
Mutation analysis originated from S protein of Indonesian SARS-CoV-2 isolates. **A** Distribution of D614G mutation from various provinces in Indonesia; **B** 3D structure visualization of SARS-CoV-2 S protein. Red dots are the location of various mutations in S protein; **C** The mapping of amino acid mutation sites in S protein of 166 Indonesian SARS-CoV-2 isolates

In this study, D614G mutation was detected in 103 Indonesian SARS-CoV-2 isolates. All the isolates were mostly from the West Java (*n* = 22), East Java (*n* = 20), Special Region of Yogyakarta (*n* = 12), Special Capital Region of Jakarta (*n* = 11), and Central Java (*n* = 7), respectively (Fig. 2A). The results also demonstrated the mapping of amino acid mutation sites in S protein of all SARS-CoV-2 isolates (Fig. 2B, C). However, this study could not find any various important novel mutations or its variants like 484K, 501Y, and 681H. The 3D visualization of structure from SARS-CoV-2 S protein was developed using WIV04 isolate. It also was marked with the red dots in every amino acid mutation of S protein occurred as mentioned previously in this study (Fig. 2B and Additional file 1).

### Molecular phylogenetic analysis

**Fig. 3:**
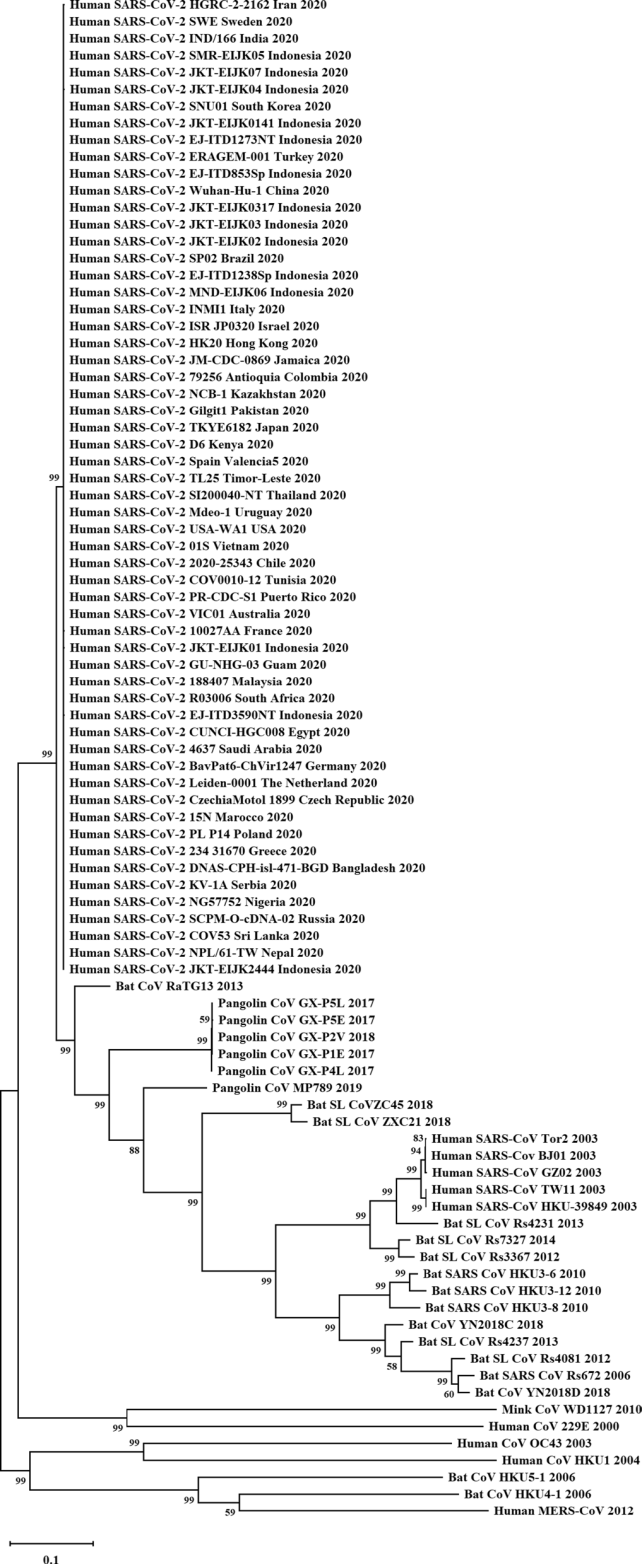
Molecular phylogenetic of SARS-CoV-2 isolates and other coronaviruses. Indonesian SARS-CoV-2 isolates and other SARS-CoV-2 isolates from various countries are grouping into a clade and closely related to CoV RATG13 from bats, CoV from pangolin, and SARS-CoV from human, respectively

This study developed a molecular phylogenetic tree and presented the relationship between Indonesian SARS-CoV-2 isolates, many isolates from various nations around the world, and the coronaviruses originating from humans, mink, bats, and pangolins (Fig. 3). Here, this research reported an advanced studied to construct the Indonesian virus isolates’ molecular phylogenetic.

## Discussion

Until the end of 2019, there were six identified coronaviruses to be causative agents of infection in humans. The seventh, SARS-CoV-2, emerged in China (Khan et al. 2020a, b; Wu et al. 2020). To date, according to the CSSE at Johns Hopkins University online website, there are more than 195 million people infected with the virus globally (Dong et al. 2020). Moreover, the reports said that human-to-human transmission has occurred and WHO has acknowledged the chance of aerosol infection (Tellier et al. 2019). Based on our study, the identification of the virus was generated from several collection methods through swabbing activities in saliva, throat, sputum, bronchoalveolar-lavage, oropharyngeal and nasopharyngeal area.

The data of 166 Indonesian SARS-CoV-2 isolates were retrieved prior to the beginning of COVID-19 vaccination program on 13 January 2021 from the database used to pool the virus samples collected from sputum, oropharyngeal and nasopharyngeal swabs by many collaborations among research centres and universities in Indonesia.

Recent updates show that GISAID EpiCoV database has acknowledged seven subtypes of SARS-CoV-2, specifically V, S, O, L, GR, GH, GV, and G clades. Significantly, the isolates from Indonesia in this study were grouped into the G, GH, GR, L, and O clades. This study also reveals those above lineages of SARS-CoV-2 isolates in Indonesia according to the accumulated data from GISAID EpiCoV database (Fig. 1). In this study, 14 lineages from 166 isolates were displayed in this study (Fig. 1). However, based on this data, there were no any novel lineages identified related to new variants, while many reports stated that novel variants of SARS-CoV-2 occurred in various countries, such as UK, Brazil, and South Africa (Ali et al. 2021; Chaillon and Smith 2021; Tegally et al. 2021; Volz et al., 2021; Nonaka et al. 2021). These novel variants might be more transmissible and suspected to be accountable for the rise of COVID-19 patient numbers in those countries (Tegally et al. 2021; Volz et al., 2021).

Meanwhile, S protein mediates the entry and membrane fusion of the new virus and is the main target for many studies of antiviral drugs and vaccines (Jean et al. 2020; Syahniar et al. 2020). S1 and S2 are the two domains of the virus S protein. S1 is conscientious for binding to host cellular receptors. Besides the efficacy of several therapies which include disrupting protease inhibitors, small RNAs, neutralizing antibodies, fusion blockers, S protein inhibitors, ACE2 blockers, however, the in vitro studies on S protein inhibitors have been unsatisfactory (Yin 2020). Many methods have been employed to produce vaccines using S protein as an antigen (Normalina et al. 2020; Watanabe et al. 2020).

Scientists have demonstrated that mutations occur in the virus genome globally (Ansori et al. 2020; Benvenuto et al. 2020; Joob and Wiwanitkit 2020; Phan 2020). Previously, Phan et al. performed a genetic analysis in 86 virus genomes and reported many mutations. One of the most important mechanisms proposed for the evolution of viruses in nature is nucleotide substitution (Phan 2020). Yadav et al. (2020) also reported a study to analyze the first two virus isolates from India, while Garcés-Ayala et al. (2020) who conducted a study with the reference sequence for fully describing the novel SARS-CoV-2 complete genome in Mexico. Khailany et al. (2020) successfully retrieved 94 SARS-CoV-2 genomes and checked the molecular variation between them. Furthermore, Kim et al. (2020) revealed that the quick transmission and infectivity of the virus correlated with specific mutations in the genome. This study reported various S protein mutations such as A222, S477, D614, Q677, and so on (Fig. 2). Further research was highly considered that S protein mutations to affect vaccination program worldwide (Le Page 2021; Xie et al. 2021; Zhang et al. 2020).

Besides, recent publications show that one of the most notable amino acid mutations is D614G (Korber et al. 2020; Nidom et al. 2020a, b). Based on these recent studies, the virus virulence and the increase of viral loads in COVID-19 patients characterize the occurrence of D614G mutation (Korber et al. 2020; Zhang et al. 2020), while, based on the current available information, the infectivity as well as the receptor binding, fusion activation, or ADE enhancement can be influenced by D614G mutation in several ways (Ulrich et al. 2020; Wang and Zand. 2020; Nidom et al. 2020a). An antibody escape is considered as another mutation mechanism like the upcoming form of D614G which can be accelerated by an antigenic drift. If the sensitivity of neutralizing antibody can be affected by D614G mutation in SARS-CoV-2 or vice versa, then the ADE activity also can be monitored in the SARS-CoV study; thus, D614G can be considered as an intermediate antibody escape which puts people to be more vulnerable for second infections (Cloutier et al. 2020; Zhang et al. 2020; Nidom et al. 2020a).

A study by Zhang et al. on D614G mutation which discovered that S1 residue 614 is in a close proximity to S2 domain. An altered release or shedding of S1 domain after cleavage at S1/S2 junction might be displayed by the ratio between S1 and S2 domains in the virion. Glycine amino acid found at residue 614 of S protein G614 secures the interaction between S1 and S2 domains and limits S1 shedding. D614G mutation has been previously speculated in raising an open configuration of S protein that is more advantageous to ACE2 association (Zhang et al. 2020). Therefore, SARS-CoV-2 S protein D614G mutation is highly believed in promoting the virion spike density and infectivity and it is also highly speculated that this mutation might be influence further mutations.

Previous studies on the molecular phylogenetic tree revealed that the relationship of SARS-CoV-2 and other Coronaviridae is based on the four structural protein genes. In accordance with this, SARS-CoV-2 is considered to be the closest to *Rhinolophus affinis* coronavirus RaTG13 and followed by pangolin coronavirus (Andersen et al. 2020). Thus, Malayan pangolin is assumed as the intermediate host before infecting to humans (Tu et al. 2020). In addition, the previous study also reported that the type of mutation emerged in the virus isolates were originated from canine, environment, *Felis catus*, mice, *Mustela lutreola*, and *Panthera tigris jacksoni* (Nidom et al. 2020a) (Fig. 3). It further advised in promoting the surveillance researches to be implemented in many mammals in their native habitats including bats and pangolins, especially in East Asia; thus, the risk of the forthcoming zoonotic diseases can be well-predicted.

Compared to most other microorganisms, the rates of RNA viruses’ mutations are much higher (Chen and Chen. 2020). An elevated mutation rate can lead to an increase in virulence and a high potential for adaptive evolution (Chen and Chen 2020; Khan et al. 2020a, b; Wang et al. 2020). This capability boosts the chance of zoonotic viral pathogens to establish human-to-human transmission and permits them to enhance their virulence (Wang et al. 2020).

This study provides the fundamental data for accomplished studies into the medication and prevention of COVID-19. Indonesian SARS-CoV-2 genomic data extraction would be valuable in vaccine construction and options in medication. In fact, mining the data from the Indonesian SARS-CoV-2 variants and molecular epidemiology could enable the mapping of its origin and the tracking of its transmission (Setiawaty et al. 2020). In line with this, the sequence investigation performs an important role in viral surveillance, public health policy problems, and host identification (Álvarez-Díaz et al. 2020; Setiawaty et al. 2020). Thus, high-speed detection of mutations from the Indonesian SARS-CoV-2 is mandatory in the unlocking to the COVID-19 pandemic in Indonesia.

As the availability of COVID-19 vaccine is limited, not many people can access it. In countries that have not implemented large-scale active case testing and isolation, controlling the spread of the virus can be very challenging. In this case, transmission suppression relies primarily on the community adherence to non-pharmacological strategies such as social distancing, the mandatory mask using, and hand washing (Bedford et al. 2020; Güner et al. 2020; Lewnard and Lo 2020; Qian and Jiang 2020). Inevitably, as the result of SARS-CoV-2 outbreaks, many countries declared medical emergency which led to the economic emergency since those countries enforcing limited or strict mobility both regionally and nationally (Ahmad et al. 2020; Hiscott et al. 2020; Palacios Cruz et al. 2021). Therefore, regulating and containing further transmission of COVID-19 is a fundamental move to discover the characteristics of SARS-CoV-2 genome and constitute the systems for observing SARS-CoV-2 during this pandemic. The recognition of genotypes related to temporal infectious clusters and specific geographic areas suggests that the employment of genomic data is highly recommended in observing and tracking the further spreading of SARS-CoV-2. Researchers might be able to introduce the origin of a specific variant and observe the virus transmission by acknowledging the specific SARS-CoV-2 variants and connecting them using a molecular epidemiology approach. Ergo, it can be argued that this study might become an important tool in regulating the COVID-19 pandemic in Indonesia.

## Conclusion

In conclusion, this study successfully identified the full-length genome mutation analysis of 166 Indonesian SARS-CoV-2 isolates. This study helps in observing the spread of the COVID-19 transmission. However, we proposed that the epidemiological surveillance and genomics studies might be improved on COVID-19 pandemic in Indonesia.

## Supplementary Information

**Additional file 1**. Table of 166 Indonesian SARS-CoV-2 Isolates and Its Mutations.

## Data Availability

All data on which abstracted of the study have been drawn are presented in the main manuscript.

## Abbreviations

ACE2: Angiotensin-converting enzyme 2
ADE: Antibody-dependent enhancement
COVID-19: Coronavirus disease 2019
CSSE: Center for Systems Science and Engineering
GISAID: Global Initiative on Sharing All Influenza Data
RNA: Ribonucleic acid
S: Spike
SARS-CoV-2: Severe acute respiratory syndrome coronavirus 2
WHO: World Health Organization
