# Supplementary material for: Phylogenetic and full-length genome mutation analysis of SARS-CoV-2 in Indonesia prior to COVID-19 vaccination program in 2021
Source: Bull Natl Res Cent. 2021 Nov 21;45(1):200. doi: 10.1186/s42269-021-00657-0 (PMC8606223; doi:10.1186/s42269-021-00657-0)
Supplement: Supplementary file 1 — Additional file 1. Table of 166 Indonesian SARS-CoV-2 Isolates and Its Mutations. [file 42269_2021_657_MOESM1_ESM.docx]

**Supplementary Material**

**Supplement 1**

**Table of 166 Indonesian SARS-CoV-2 Isolates and Its Mutations.**

| **No** | **Isolate** | **Province** | **Gene** | **Mutation** |
| --- | --- | --- | --- | --- |
| 1 | JI-ITDua-5392NTv | East Java | NSP12 | P227L, P323L |
|  |  |  | Spike | D614G |
|  |  |  | NS3 | Q57H |
| 2 | JI-ITDua-5748NTv | East Java | NSP12 | P227L, P323L |
|  |  |  | NSP14 | V14L |
|  |  |  | Spike | D614G |
|  |  |  | NS3 | Q57H |
| 3 | JI-ITDua-998NTv | East Java | NSP12 | A399V |
| 4 | JI-ITDua-1006NTv | East Java | NSP12 | A399V |
| 5 | JI-ITDua-4134NTv | East Java | NSP12 | P227L, P323L |
|  |  |  | Spike | D614G |
|  |  |  | NS3 | Q57H |
| 6 | JI-ITDua-12323Nvv | East Java | NSP5 | L220F |
|  |  |  | NSP12 | P227L, P323L |
|  |  |  | Spike | D614G |
|  |  |  | NS3 | Q57H |
| 7 | JI-ITDua-6033NTv | East Java | NSP3 | L198F |
|  |  |  | NSP12 | P323L, V354L |
|  |  |  | Spike | D614G |
|  |  |  | NS3 | Q57H |
| 8 | JI-ITDua-2858NTv | East Java | NSP12 | P227L, P323L |
|  |  |  | Spike | D614G, Q677H |
|  |  |  | NS3 | Q57H |
| 9 | JI-ITDua-1609Nv | East Java | NSP12 | P323L |
|  |  |  | Spike | D614G |
|  |  |  | NS3 | Q57H |
| 10 | JI-ITDua-28257NT | East Java | NSP2 | Y16N |
|  |  |  | NSP12 | P227L, P323L |
|  |  |  | Spike | D614G |
|  |  |  | NS3 | Q57H, S220I |
|  |  |  | NS7b | E39stop |
|  |  |  | N | T205I |
| 11 | JB-WJHL-ITB-R120975 | West Java | NSP2 | E565A |
|  |  |  | NSP4 | A231V |
|  |  |  | NSP12 | M110V, P323L |
|  |  |  | Spike | A222V, D614G |
|  |  |  | N | R203K, G204R |
| 12 | KS-NIHRD-PME4901 | South Kalimantan | NSP6 | L260F |
|  |  |  | NSP12 | P323L |
|  |  |  | NSP13 | A520V |
|  |  |  | Spike | D614G |
|  |  |  | NS3 | Q57H |
|  |  |  | NS6 | N47I |
|  |  |  | NS7b | L4F |
|  |  |  | N | S187L |
| 13 | JB-TFRIC19-R46646 | West Java | NSP2 | T601X |
|  |  |  | NSP4 | Y335X |
|  |  |  | NSP5 | A234X |
|  |  |  | NSP6 | L37X |
|  |  |  | NSP12 | P227X |
|  |  |  | NSP14 | P443X, G480X, A482X, C484X, R485X |
|  |  |  | NSP16 | A178X, M270I |
|  |  |  | Spike | V267X, T302X, K310X, D614X |
|  |  |  | NS6 | F2X |
|  |  |  | NS7a | L56X, Q62X, A66X, P84X, S98X, I100X |
|  |  |  | N | S33X, S202G |
| 14 | JB-TFRIC19-R50090 | West Java | NSP3 | P822L, A1711X |
|  |  |  | NSP4 | A307X |
|  |  |  | NSP5 | K90R |
|  |  |  | NSP12 | P323L, C464X, A656X |
|  |  |  | NSP13 | M576X |
|  |  |  | NSP14 | A482X |
|  |  |  | Spike | P295X, L296X, S297X, E309X, T315X, R457X, D614G |
|  |  |  | NS3 | Q57X |
|  |  |  | NS7a | A50X, A66X |
|  |  |  | N | S193I |
| 15 | JB-NIHRDC-0056536 | West Java | NSP3 | L1328F |
|  |  |  | NSP12 | P323L |
|  |  |  | Spike | D614G |
|  |  |  | NS3 | A72S |
|  |  |  | N | R203K, G204R, P365S |
| 16 | SU-NIHRDC-0045870 | North Sumatra | NSP2 | I58V |
|  |  |  | NSP3 | A58T |
|  |  |  | NSP4 | A380V |
|  |  |  | NSP6 | L37F |
|  |  |  | NSP12 | P323L |
|  |  |  | Spike | D614G |
|  |  |  | NS3 | Q57H |
|  |  |  | NS8 | V62L |
|  |  |  | N | A398V |
| 17 | AC-NIHRD-C0057926 | Aceh | NSP3 | A58T, L1244F |
|  |  |  | NSP7 | S25L |
|  |  |  | NSP12 | P323L |
|  |  |  | Spike | D614G |
|  |  |  | NS3 | Q57H |
|  |  |  | NS8 | V62L |
| 18 | JT-UNS-18_39701 | Central Java | NSP2 | E53X, H54X, E55X, H56X, E57X, I58X, A59X, W60X, Y61X |
|  |  |  | NSP3 | P822L, L895X, I896X, L897X, A898X, Y899X, C900X, N901X, K902X, T903X, V904X, G905X |
|  |  |  | NSP8 | K36X, K37X, L38X, K39X, K40X, S41X |
|  |  |  | NSP12 | P323L, Y346X, H347X, F348X, R349X, E350X, L351X, G352X, V353X, V354X, M615X, G616X, W617X, D618X |
|  |  |  | NSP13 | E365D |
|  |  |  | NSP15 | F258S |
|  |  |  | NSP16 | I237X, Q238X, L239X, S240X, S241X, Y242X |
|  |  |  | Spike | D614G, S939X, S940X, T941X, A942X, S943X |
|  |  |  | NS3 | Q57H, Y141X, D142X, A143X, N144X, Y145X, F146X, L147X, C148X, W149X, H150X, T151X, N152X, C153X, Y154X, D155X, Y156X, C157X, I158X, P159X, Y160X, N161X, S162X, V163X, T164X, S165X, S166X, I167X, V168X, I169X, T170X, S171X, G172X, D173X, G174X, T175X, T176X, S177X, P178X, I179X, T223I |
|  |  |  | E | Y42X, C43X, C44X |
|  |  |  | N | T247X, K248X, K249X, S250X, A251X |
| 19 | JI-ITDua-4437NTv | East Java | NSP12 | P323L |
|  |  |  | Spike | D614G |
|  |  |  | NS3 | Q57H |
| 20 | JI-ITDua-5235NTv | East Java | NSP12 | P323L, A529V |
|  |  |  | Spike | D614G |
|  |  |  | NS3 | Q57H |
| 21 | BT-SHSIU-01-4941 | Banten | NSP3 | D174Y, P822L |
|  |  |  | NSP6 | T103N |
|  |  |  | NSP12 | P323L, T769I |
|  |  |  | Spike | L5F, D614G |
|  |  |  | NS3 | Q57H, G172C |
|  |  |  | NS7a | Q94H |
|  |  |  | N | R203M |
| 22 | BT-SHSIU-01-4920 | Banten | NSP2 | E490K |
|  |  |  | NSP3 | P822L |
|  |  |  | NSP4 | S386F |
|  |  |  | NSP5 | K12R |
|  |  |  | NSP10 | P8S |
|  |  |  | NSP12 | T248I, P323L |
|  |  |  | Spike | V213A, D614G |
|  |  |  | NS3 | Q57H |
|  |  |  | N | A119S, S193I |
| 23 | JB-WJHL-ITB-L18952 | West Java | NSP2 | V628I |
|  |  |  | NSP4 | P187S |
|  |  |  | NSP12 | P323L |
|  |  |  | Spike | D614G |
|  |  |  | N | P67S, R203K, G204R |
| 24 | JK-NIHRD-IST002 | Jakarta | NSP3 | P822L |
|  |  |  | NSP8 | V34F |
|  |  |  | NSP12 | P323L, G596S |
|  |  |  | Spike | D614G |
|  |  |  | NS3 | Q57H |
|  |  |  | N | S193I |
| 25 | JK-NIHRD-PME1264 | Jakarta | NSP12 | P323L |
|  |  |  | Spike | D614G |
|  |  |  | N | R203K, G204R |
| 26 | JB-WJHL-ITB-A02050 | West Java | NSP1 | L149R |
|  |  |  | NSP2 | I273T |
|  |  |  | NSP3 | T181I, N1220S |
|  |  |  | NSP4 | G309C |
|  |  |  | NSP10 | A32V |
|  |  |  | NSP12 | P323L |
|  |  |  | Spike | D614G |
|  |  |  | N | R195S, R203K, G204R |
| 27 | JB-WJHL-ITB-A03267 | West Java | NSP3 | T1036I |
|  |  |  | NSP12 | D109N, P323L |
|  |  |  | Spike | D614G |
|  |  |  | E | L21F |
|  |  |  | N | R203K, G204R |
| 28 | JB-WJHL-ITB-R104416 | West Java | NSP12 | P323L |
|  |  |  | Spike | D614G |
|  |  |  | NS8 | T87I |
|  |  |  | N | R203K, G204R |
| 29 | JB-WJHL-ITB-R109969 | West Java | NSP1 | K141 (del), S142 (del), F143 (del) |
|  |  |  | NSP12 | P323L |
|  |  |  | NSP15 | T33I |
|  |  |  | Spike | D614G |
|  |  |  | N | R203K, G204R |
| 30 | JB-WJHL-ITB-R119840 | West Java | NSP12 | P323L |
|  |  |  | Spike | Q613H, D614G |
|  |  |  | NS8 | T26I |
|  |  |  | N | R203K, G204R, T332I |
| 31 | YO-UGM-10007 | Yogyakarta | NSP3 | P822L, A1179V, F1354C, P1665L |
|  |  |  | NSP12 | P323L |
|  |  |  | Spike | D614G, K811I |
|  |  |  | NS3 | Q57H, T151I |
| 32 | YO-UGM-48651 | Yogyakarta | NSP12 | P227L, P323L |
|  |  |  | Spike | V83L, D614G, Q677H |
|  |  |  | NS3 | Q57H, D222Y |
|  |  |  | N | M234I |
| 33 | BT-SHSIU-01-3610 | Banten | NSP2 | E490K |
|  |  |  | NSP3 | P822L |
|  |  |  | NSP4 | S386F |
|  |  |  | NSP5 | K12R |
|  |  |  | NSP10 | P8S |
|  |  |  | NSP12 | T248I, P323L |
|  |  |  | Spike | V213A, D614G |
|  |  |  | NS3 | Q57H |
|  |  |  | N | A119S, S193I |
| 34 | YO-UGM-10003 | Yogyakarta | NSP2 | A247V |
|  |  |  | NSP3 | P822L |
|  |  |  | NSP5 | K12R |
|  |  |  | NSP12 | T248I, P323L |
|  |  |  | Spike | V213A, D614G |
|  |  |  | NS3 | Q57H |
|  |  |  | N | A119S, S193I |
| 35 | YO-UGM-10002 | Yogyakarta | NSP2 | T256I, S494X, V495X, Q496X, T497X, T629X, N630X, N631X, T632X, F633X |
|  |  |  | NSP3 | P822L |
|  |  |  | NSP5 | K12R |
|  |  |  | NSP12 | T248I, P323L |
|  |  |  | Spike | V213A, D614G |
|  |  |  | NS3 | Q57H |
|  |  |  | N | A119S, S193I |
| 36 | YO-UGM-10005 | Yogyakarta | NSP2 | T256I, Q321K |
|  |  |  | NSP3 | P822L |
|  |  |  | NSP12 | P323L, H892Y |
|  |  |  | Spike | L5F, D614G |
|  |  |  | NS3 | Q57H |
| 37 | YO-UGM-10006 | Yogyakarta | NSP2 | T256I, Q321K |
|  |  |  | NSP3 | P822L |
|  |  |  | NSP12 | P323L, H892Y |
|  |  |  | Spike | L5F, D614G |
|  |  |  | NS3 | Q57H |
| 38 | YO-UGM-107727 | Yogyakarta | NSP5 | P184S |
|  |  |  | NSP12 | P323L |
|  |  |  | NSP15 | H337Y |
|  |  |  | Spike | D614G |
|  |  |  | N | R195S, R203K, G204R |
| 39 | JT-UGM-47906 | Central Java | NSP2 | A205V |
|  |  |  | NSP12 | P323L |
|  |  |  | NSP16 | Y222C |
|  |  |  | Spike | D614G |
|  |  |  | N | P199S, R203K, G204R |
| 40 | JK-NIHRD-MI43269 | Jakarta | NSP12 | P323L |
|  |  |  | NSP15 | K12N |
|  |  |  | Spike | D614G |
|  |  |  | N | R203K, G204R |
| 41 | JK-NIHRD-MI52948 | Jakarta | NSP12 | P323L |
|  |  |  | NSP13 | S589C |
|  |  |  | Spike | P26S, D614G |
|  |  |  | NS7a | R89I |
|  |  |  | N | R203K, G204R |
| 42 | JT-UGM-48660 | Central Java | NSP3 | P822L |
|  |  |  | NSP5 | K12R |
|  |  |  | NSP12 | T248I, P323L |
|  |  |  | NSP13 | V169F |
|  |  |  | Spike | V213A, D614G |
|  |  |  | NS3 | Q57H |
|  |  |  | N | A119S, S193I |
| 43 | JT-UGM-47964 | Central Java | NSP3 | T1198K |
|  |  |  | NSP6 | L37F |
|  |  |  | NSP8 | A21T |
|  |  |  | NSP9 | L42F |
|  |  |  | NSP12 | A97V, M906V |
|  |  |  | NSP13 | T153I |
|  |  |  | Spike | W258R, D614G |
|  |  |  | N | P13L |
| 44 | SA-EIJK51 | North Sulawesi | NSP3 | T1198K |
|  |  |  | NSP6 | L37F |
|  |  |  | NSP12 | A97V |
|  |  |  | Spike | D614G |
|  |  |  | N | P13L |
| 45 | SA-EIJK52 | North Sulawesi | NSP3 | T1198K |
|  |  |  | NSP6 | L37F |
|  |  |  | NSP12 | A97V |
|  |  |  | Spike | D614G |
|  |  |  | N | P13L |
| 46 | SA-EIJK53 | North Sulawesi | NSP3 | T1198K |
|  |  |  | NSP6 | L37F |
|  |  |  | NSP12 | A97V |
|  |  |  | Spike | D614G |
|  |  |  | N | P13L |
| 47 | SA-EIJK54 | North Sulawesi | NSP3 | T1198K |
|  |  |  | NSP4 | D161G |
|  |  |  | NSP6 | L37F |
|  |  |  | NSP12 | A97V |
|  |  |  | NSP13 | T153I |
|  |  |  | Spike | D614G |
|  |  |  | N | P13L |
| 48 | YO-UGM-00061 | Yogyakarta | NSP3 | P822L, T1022I |
|  |  |  | NSP12 | P323L |
|  |  |  | Spike | D614G |
|  |  |  | NS3 | Q57H |
| 49 | YO-UGM-10001 | Yogyakarta | NSP2 | A247V |
|  |  |  | NSP3 | P822L |
|  |  |  | NSP5 | K12R |
|  |  |  | NSP12 | T248I, P323L |
|  |  |  | Spike | V213A, D614G |
|  |  |  | NS3 | Q57H |
|  |  |  | N | A119S, S193I |
| 50 | YO-UGM-10004 | Yogyakarta | NSP3 | V299A, P822L |
|  |  |  | NSP4 | A231V |
|  |  |  | NSP12 | P323L |
|  |  |  | NSP13 | T127I |
|  |  |  | NSP14 | P203L |
|  |  |  | Spike | D614G |
|  |  |  | NS3 | Q57H |
| 51 | JK-EIJK34 | Jakarta | NSP3 | K1804N |
|  |  |  | NSP12 | A399V |
| 52 | BT-EIJK36 | Banten | NSP2 | T223I |
|  |  |  | NSP3 | Q1884H |
|  |  |  | NSP5 | A234V |
|  |  |  | NS8 | P30Q |
| 53 | JB-EIJK41 | West Java | NSP12 | D92N |
|  |  |  | N | G179S |
| 54 | JK-EIJK42 | Jakarta | NSP2 | K500N |
| 55 | JK-EIJK45 | Jakarta | NSP3 | T1198K |
|  |  |  | NSP6 | L37F |
|  |  |  | NSP12 | A97V |
|  |  |  | N | P13L, R209I |
| 56 | JK-EIJK46 | Jakarta | NSP2 | S122P |
|  |  |  | NSP12 | D92N |
|  |  |  | NSP13 | S80G |
|  |  |  | NS6 | T10I |
| 57 | JK-EIJK47 | Jakarta | NSP12 | D60N, A399V |
|  |  |  | Spike | V70F |
| 58 | JK-EIJK48 | Jakarta | NSP5 | S254F |
|  |  |  | NSP6 | L37F |
|  |  |  | NS3 | A72P |
|  |  |  | NS6 | D6Y |
| 59 | JK-EIJK49 | Jakarta | NSP3 | P822L |
|  |  |  | NSP12 | P323L, G596S |
|  |  |  | Spike | D614G |
|  |  |  | NS3 | Q57H |
|  |  |  | N | S193I |
| 60 | SA-EIJK50 | North Sulawesi | NSP2 | Y2C, T85I |
|  |  |  | NSP12 | P323L |
|  |  |  | Spike | D614G |
| 61 | PP-EIJK-11 | Papua | NO MUTATION | |
| 62 | SA-EIJK-12 | North Sulawesi | N | A381V |
| 63 | JK-NIHRD-C0027475 | Jakarta | NSP3 | P822L, E906X, L907X, R1371X, I1372X, K1373X, A1374X, S1375X, T1459X, I1460X, A1461X, S1494X, K1497X, M1593X, C1594X, R1597X, P1719X, A1803X, V1806X, S1807X |
|  |  |  | NSP4 | Y82X, T83X, N84X, K86X, Y397X |
|  |  |  | NSP6 | L37F |
|  |  |  | NSP12 | P323L, G596S |
|  |  |  | NSP14 | S56X, M57X |
|  |  |  | Spike | D614G, A1080X, A1087X, H1088X, A1174X |
|  |  |  | NS3 | Q57H |
|  |  |  | N | S193I, Q289H |
| 64 | PA-EIJK09 | Papua | NSP12 | A399V |
| 65 | SA-EIJK13 | North Sulawesi | NSP12 | R889K |
| 66 | YO-EIJK15 | Yogyakarta | NSP3 | V598F, A898P |
|  |  |  | NSP12 | P868L |
|  |  |  | NSP13 | E591K |
| 67 | JB-EIJK16 | West Java | NSP3 | T1198K |
|  |  |  | NSP6 | L37F |
|  |  |  | NSP12 | A97V |
|  |  |  | Spike | G1124V |
|  |  |  | N | P13L |
| 68 | JK-EIJK19 | Jakarta | NSP6 | M192T |
|  |  |  | E | K63R |
| 69 | JK-EIJK20 | Jakarta | NSP2 | T474I |
|  |  |  | NSP4 | H313Y |
|  |  |  | NSP12 | A399V |
| 70 | JK-EIJK24 | Jakarta | NSP12 | A399V |
| 71 | PA-NIHRD-PME4306 | Papua | NSP3 | D714G, P968H |
|  |  |  | NSP13 | H290Y |
| 72 | JB-NIHRD-PME4992 | West Java | NSP3 | P822L |
|  |  |  | NSP12 | P323L, G596S |
|  |  |  | Spike | D614G |
|  |  |  | NS3 | Q57H |
|  |  |  | N | S193I |
| 73 | EJ-ITD-3601NT | East Java | NSP3 | T1198K |
|  |  |  | NSP6 | L37F |
|  |  |  | NSP12 | A97V |
|  |  |  | M | S214I |
|  |  |  | N | P13L |
| 74 | JI-EIJK-10 | East Java | NO MUTATION | |
| 75 | JK-EIJK-23 | Jakarta | NSP1 | N160S |
| 76 | JK-EIJK-32 | Jakarta | NSP12 | A399V |
| 77 | JK-EIJK-38 | Jakarta | NSP2 | E595G |
|  |  |  | NSP6 | V114A |
|  |  |  | NSP12 | P323L |
|  |  |  | Spike | D614G |
|  |  |  | NS3 | Q57H |
| 78 | JK-EIJK-39 | Jakarta | Spike | C1254F |
| 79 | JK-EIJK-40 | Jakarta | NSP4 | S432N |
|  |  |  | NSP12 | D92N |
|  |  |  | NSP16 | M247I |
| 80 | JK-EIJK-43 | Jakarta | NSP5 | S254F |
|  |  |  | NSP6 | L37F |
|  |  |  | NS3 | A72P |
|  |  |  | NS6 | D6Y |
| 81 | KT-NIHRD-PME4206 | Central Kalimantan | NSP4 | S395I |
|  |  |  | NSP6 | N40Y |
|  |  |  | NSP12 | P323L |
|  |  |  | NSP13 | V209I |
|  |  |  | Spike | D614G |
|  |  |  | NS3 | Q57H |
| 82 | BA-NIHRD-PME3926 | Bali | NSP12 | P323L |
|  |  |  | NSP15 | T33I |
|  |  |  | Spike | D614G |
|  |  |  | N | R203K, G204R |
| 83 | JK-NIHRD-MI41613 | Jakarta | NSP3 | K1693N |
|  |  |  | NSP12 | P323L |
|  |  |  | Spike | D614G, E654Q |
|  |  |  | N | R203K, G204R |
| 84 | JK-NIHRD-MI52885 | Jakarta | NSP2 | S305G, F368L |
|  |  |  | NSP3 | P968L, S1314G |
|  |  |  | NSP12 | P323L |
|  |  |  | Spike | D614G |
|  |  |  | NS3 | V13L |
|  |  |  | N | R203K, G204R |
| 85 | PA-NIHRD-C0710717 | Papua | NSP3 | D714G |
|  |  |  | N | R209I |
| 86 | PA-NIHRD-C0710684 | Papua | NSP3 | D714G |
| 87 | JK-NIHRD-MI2851 | Jakarta | NSP12 | P323L |
|  |  |  | NSP15 | N177D |
|  |  |  | NSP16 | V194I |
|  |  |  | Spike | D614G |
|  |  |  | N | R203K, G204R |
| 88 | JK-NIHRD-MI52946 | Jakarta | NSP12 | P323L |
|  |  |  | NSP13 | S589C |
|  |  |  | Spike | P26S, D614G |
|  |  |  | NS7a | R89I |
|  |  |  | N | R203K, G204R |
| 89 | KS-NIHRD-PME4900 | South Kalimantan | NSP6 | L260F |
|  |  |  | NSP12 | P323L |
|  |  |  | NSP13 | A520V |
|  |  |  | Spike | D614G |
|  |  |  | NS3 | Q57H |
|  |  |  | NS6 | N47I |
|  |  |  | NS7b | L4F |
|  |  |  | N | S187L |
| 90 | JI-NIHRD-PME5011 | East Java | NSP2 | T44I |
|  |  |  | NSP3 | G307D, V469M |
|  |  |  | NSP12 | P323L |
|  |  |  | NSP13 | L43F |
|  |  |  | Spike | D614G |
|  |  |  | NS3 | Q57H |
|  |  |  | NS7b | C41F |
|  |  |  | N | S194L |
| 91 | JI-ITD-150Sp | East Java | NO MUTATION | |
| 92 | JI-ITD-17398NT | East Java | NSP3 | S1670F |
|  |  |  | NSP12 | P227L, P323L |
|  |  |  | Spike | T22I, A67V, D614G |
|  |  |  | NS3 | Q57H |
|  |  |  | E | P71S |
|  |  |  | NS8 | A51V |
| 93 | JI-ITD-1273V | East Java | NSP3 | E1271D |
|  |  |  | NSP13 | R392H |
|  |  |  | Spike | A352S |
| 94 | JI-ITD-4859V | East Java | NSP12 | P227L, P323L |
|  |  |  | Spike | D614G |
|  |  |  | NS3 | Q57H |
| 95 | JI-ITD-7061V | East Java | NSP12 | P323L |
|  |  |  | Spike | D614G |
|  |  |  | NS3 | Q57H |
| 96 | JI-ITD-12202V | East Java | NSP12 | P227L, P323L |
|  |  |  | Spike | D614G |
|  |  |  | NS3 | Q57H |
| 97 | JI-NIHRD-PME0999 | East Java | NSP12 | P227L, P323L |
|  |  |  | Spike | D614G |
|  |  |  | NS3 | Q57H |
| 98 | JI-NIHRD-PME2054 | East Java | NSP12 | P227L, P323L |
|  |  |  | NSP14 | A360S |
|  |  |  | Spike | D614G |
|  |  |  | NS3 | Q57H |
| 99 | SU-NIHRD-PME4745 | North Sumatra | NSP3 | A58T, L1244F |
|  |  |  | NSP5 | L50F |
|  |  |  | NSP7 | S25L |
|  |  |  | NSP12 | P323L |
|  |  |  | Spike | D614G |
|  |  |  | NS3 | Q57H |
|  |  |  | NS8 | V62L |
| 100 | SU-NIHRD-PME4760 | North Sumatra | NSP3 | A58T |
|  |  |  | NSP6 | L37F, A79V |
|  |  |  | NSP12 | P323L |
|  |  |  | Spike | D614G |
|  |  |  | NS3 | Q57H |
|  |  |  | NS8 | V62L |
| 101 | JB-TFRIC19-R30535 | West Java | NSP2 | D409X |
|  |  |  | NSP12 | P323L |
|  |  |  | NSP14 | A504S |
|  |  |  | Spike | D614G |
|  |  |  | N | R203K, G204R |
| 102 | JB-TFRIC19-R48562 | West Java | NSP2 | I273X |
|  |  |  | NSP12 | P227X, P323L |
|  |  |  | NSP14 | L280X, M500X |
|  |  |  | Spike | D614G |
|  |  |  | NS3 | Q57X |
|  |  |  | M | A38X |
|  |  |  | N | R203X, G204X |
| 103 | JB-TFRIC19-R4975 | West Java | NSP2 | T85I |
|  |  |  | NSP12 | P323L |
|  |  |  | Spike | R214L, D614G |
|  |  |  | NS3 | Q57H |
| 104 | JB-TFRIC19-R49542 | West Java | NSP3 | A450S |
|  |  |  | NSP12 | P323L, A529V |
|  |  |  | NSP14 | A281S |
|  |  |  | Spike | R158S, D614G |
|  |  |  | NS3 | Q57H, T223I |
| 105 | JB-TFRIC19-R49344 | West Java | NSP4 | A446V |
|  |  |  | NSP12 | P227L, P323L, A529V |
|  |  |  | NSP16 | K160R |
|  |  |  | Spike | D614G, Q677H |
|  |  |  | NS3 | Q57H, V255X, V256X |
| 106 | JB-TFRIC19-R48875 | West Java | NSP3 | T64I, P822L, K1596X, C1641F |
|  |  |  | NSP12 | P323L, G596S |
|  |  |  | NSP15 | V127F |
|  |  |  | Spike | D614G |
|  |  |  | NS3 | Q57H |
|  |  |  | N | S193I |
| 107 | JB-TFRIC19-R53817 | West Java | NSP2 | I273T |
|  |  |  | NSP12 | P227L, P323L |
|  |  |  | NSP14 | M500I |
|  |  |  | Spike | D614G |
|  |  |  | NS3 | Q57H |
| 108 | JB-TFRIC19-R49544 | West Java | NSP1 | L88X |
|  |  |  | NSP12 | T85X, P227L, P323L |
|  |  |  | Spike | T95I, D614G, Q677H |
|  |  |  | NS3 | Q57H, D222Y |
| 109 | EJ-ITD-8402NT | East Java | NSP12 | P323L |
|  |  |  | Spike | D614G |
|  |  |  | NS3 | Q57H |
| 110 | JI-ITD-136N | East Java | NSP15 | Y225C |
| 111 | SA-EIJK-06 | North Sulawesi | NO MUTATION | |
| 112 | JK-EIJK-07 | Jakarta | Spike | C1254F |
| 113 | JB-TFRIC19-L4173 | West Java | NSP1 | V5X, D48X, G49X, Q66X, V121X, L122X, L123X, R124X, K125X, N126X, G127X, G133X, E159X |
|  |  |  | NSP2 | R46X, E63X, P96X |
|  |  |  | NSP3 | V325X, V950I, L1903X |
|  |  |  | NSP6 | L37F, A117X |
|  |  |  | NSP12 | Y69X, T85X, I145X, P227L, P323L |
|  |  |  | NSP15 | V172I |
|  |  |  | Spike | N185Y, D614G, V987X |
|  |  |  | NS3 | Q57H |
|  |  |  | N | D98X, D103X, R107X, W108X, R259X |
| 114 | YO-UGM-202449 | Yogyakarta | NSP3 | P822L |
|  |  |  | NSP12 | P323L |
|  |  |  | Spike | D614G |
|  |  |  | NS3 | Q57H |
| 115 | YO-UGM-200927 | Yogyakarta | NSP5 | M49I |
| 116 | YO-UGM-781481 | Yogyakarta | NSP3 | P679S |
|  |  |  | NSP12 | P323L, A656S |
|  |  |  | NSP13 | M576I |
|  |  |  | Spike | D614G |
|  |  |  | NS3 | A54V, Q57H, A99S |
|  |  |  | N | Q160R |
| 117 | TNG-LIPI001 | Banten | NSP3 | L27F |
|  |  |  | NSP4 | I158X |
|  |  |  | NSP12 | P227L, P323L |
|  |  |  | NSP13 | L7X |
|  |  |  | NSP15 | F15S |
|  |  |  | Spike | T22P, S477I, D614G, P1140X |
|  |  |  | NS3 | Q57H, L106F |
|  |  |  | N | P20L |
| 118 | JT-UGM-202538 | Central Java | NSP3 | P822L |
|  |  |  | NSP12 | P323L |
|  |  |  | Spike | D614G |
|  |  |  | NS3 | Q57H |
|  |  |  | NS7a | H73Y |
| 119 | JB-TFRIC19-R24754 | West Java | NSP2 | I273T |
|  |  |  | NSP12 | P227L, P323L |
|  |  |  | NSP14 | M500I |
|  |  |  | Spike | D614G |
|  |  |  | NS3 | Q57H |
| 120 | JB-TFRIC19-R47609 | West Java | NSP2 | G339S |
|  |  |  | NSP5 | N274X |
|  |  |  | NSP6 | G277S |
|  |  |  | NSP12 | P227L, P323L |
|  |  |  | Spike | D614G |
|  |  |  | NS3 | Q57H |
| 121 | JI-ITD-853Sp | East Java | NO MUTATION | |
| 122 | JI-ITD-3590NT | East Java | NSP12 | P227L, P323L |
|  |  |  | Spike | S116C, D614G, Q677H |
|  |  |  | NS3 | Q57H |
| 123 | JK-EIJK-01 | Jakarta | NSP12 | A399V |
|  |  |  | Spike | V622F |
| 124 | JK-EIJK-02 | Jakarta | NO MUTATION | |
| 125 | JK-EIJK-03 | Jakarta | NSP6 | Q208H |
| 126 | JK-EIJK-04 | Jakarta | NSP12 | A399V, E744D |
|  |  |  | Spike | T572I, L822F |
|  |  |  | N | K347N |
| 127 | JI-ITD-1238Sp | East Java | NSP3 | T1378X, T1379X, I1380X, A1381X, K1382X, N1383X, T1384X, V1385X, K1386X, S1387X, V1388X, G1389X, K1390X, F1391X, C1392X, L1393X, E1394X, A1395X, S1396X, F1397X, N1398X, Y1399X, L1400X, K1401X, S1402X, P1403X, N1404X, F1405X, S1406X, K1407X, L1408X, I1409X, N1410X, I1411X, I1412X, I1413X, W1414X, F1415X, L1416X, L1417X, L1418X, S1419X, V1420X, C1421X, L1422X, G1423X, S1424X, L1425X, I1426X, Y1427X, S1428X, T1429X |
|  |  |  | NSP12 | V354L, A399V, N414X, F415X, N416X |
| 128 | JI-ITD-1273NT | East Java | NSP3 | E1271D, T1379X, I1380X, K1386X, S1387X, V1388X, G1389X, K1390X |
|  |  |  | NSP13 | R392H |
|  |  |  | Spike | A352S |
| 129 | JI-ITD-3101NT | East Java | NO MUTATION | |
| 130 | KI-EIJK-05 | East Kalimantan | Spike | A672V |
| 131 | JK-EIJK-0141 | Jakarta | NSP3 | S1285F |
| 132 | JK-EIJK-0317 | Jakarta | NSP2 | I281V |
|  |  |  | NSP12 | A399V |
| 133 | JK-EIJK-2444 | Jakarta | NSP6 | L37F |
|  |  |  | Spike | T76I |
| 134 | YO-EIJK08 | Yogyakarta | NO MUTATION | |
| 135 | NT-EIJK14 | East Nusa Tenggara | NSP3 | I1683V, H1698R, H1702R, N1708D |
|  |  |  | NSP12 | A399V |
|  |  |  | Spike | E96D |
| 136 | BT-EIJK17 | Banten | NSP12 | A399V |
| 137 | JB-EIJK18 | West Java | NSP12 | A399V |
|  |  |  | Spike | T1105P |
|  |  |  | NS7a | T28I |
| 138 | BT-EIJK21 | Banten | NSP6 | A46V |
|  |  |  | NSP12 | P323L |
|  |  |  | Spike | D614G |
|  |  |  | NS3 | Q57H |
| 139 | JK-EIJK22 | Jakarta | NSP12 | A399V |
|  |  |  | Spike | N148T |
| 140 | JK-EIJK25 | Jakarta | NSP12 | A399V |
|  |  |  | N | T24I |
| 141 | NT-EIJK27 | East Nusa Tenggara | NSP3 | C1114R, G1128D |
|  |  |  | NSP12 | A399V |
|  |  |  | NSP13 | T115I |
| 142 | JK-EIJK28 | Jakarta | NSP3 | T1198K |
|  |  |  | NSP6 | L37F |
|  |  |  | NSP12 | A97V |
|  |  |  | N | P13L |
| 143 | JK-EIJK29 | Jakarta | Spike | H655Y |
| 144 | BT-EIJK30 | Banten | NSP2 | S32L |
|  |  |  | NSP4 | A457V |
|  |  |  | NS7a | I88T |
| 145 | JK-EIJK31 | Jakarta | NSP12 | A399V |
|  |  |  | NSP14 | R163C |
|  |  |  | Spike | T76I |
| 146 | JK-EIJK33 | Jakarta | Spike | C1254F |
| 147 | JK-EIJK35 | Jakarta | NSP12 | A399V |
| 148 | JK-EIJK37 | Jakarta | NSP12 | A399V |
| 149 | JK-EIJK44 | Jakarta | NSP5 | S254F |
|  |  |  | NSP6 | L37F |
|  |  |  | NS3 | A72P |
|  |  |  | NS6 | D6Y |
| 150 | NT-EIJK26 | East Nusa Tenggara | NSP12 | A399V |
|  |  |  | Spike | F347L, A348Q |
| 151 | LA-NIHRD-PME8719 | Lampung | NSP2 | C509Y |
|  |  |  | NSP3 | A58T, L1244F, C1347F |
|  |  |  | NSP12 | P323L |
|  |  |  | Spike | D614G |
|  |  |  | NS3 | Q57H |
|  |  |  | NS8 | V62L |
|  |  |  | N | G238C |
| 152 | BA-NIHRD-PME5833 | Bali | NSP2 | D144G |
|  |  |  | NSP3 | P340S |
|  |  |  | NSP12 | P323L |
|  |  |  | NSP15 | T33I |
|  |  |  | Spike | D614G |
|  |  |  | N | R203K, G204R |
| 153 | PA-NIHRD-PME8510 | West Papua | NSP2 | P181T |
|  |  |  | NSP3 | A85V, P822L |
|  |  |  | NSP4 | H36Y |
|  |  |  | NSP12 | P323L |
|  |  |  | NSP15 | V320L |
|  |  |  | Spike | D614G, Q690H |
|  |  |  | NS3 | Q57H, T89I |
|  |  |  | N | R203K |
| 154 | NB-NIHRD-PME1393 | West Nusa Tenggara | NSP2 | T256I |
|  |  |  | NSP3 | P822L |
|  |  |  | NSP12 | P323L |
|  |  |  | Spike | D614G |
|  |  |  | NS3 | Q57H |
| **155** | **KI-NIHRD-PME5218** | **East Kalimantan** | **NSP3** | **M560I** |
|  |  |  | NSP6 | F55X |
|  |  |  | NSP12 | T28X, D29X, V30X, V31X, P323L |
|  |  |  | Spike | T259I, D614G |
|  |  |  | NS3 | Q57H |
|  |  |  | NS8 | L95F |
|  |  |  | N | T379I |
| 156 | LA-NIHRD-PME1024 | Lampung | NSP12 | P323L |
|  |  |  | Spike | D614G |
|  |  |  | NS3 | Q57H |
| 157 | BE-NIHRD-PME9915 | Bengkulu | NSP3 | A1736V |
|  |  |  | NSP6 | L37F |
|  |  |  | NSP12 | P323L |
|  |  |  | Spike | D614G |
|  |  |  | NS3 | I20T, Q57H |
|  |  |  | NS8 | A51V, A65S |
|  |  |  | N 2 | S194L, D377Y |
| 158 | JI-NIHRD-PME9750 | East Java | NSP2 | S263P |
|  |  |  | NSP3 | V469M, T749I |
|  |  |  | NSP12 | P323L |
|  |  |  | Spike | D614G |
|  |  |  | NS3 | L53F, Q57H, S171L |
|  |  |  | E | S68Y |
|  |  |  | NS8 | P93L |
|  |  |  | N | L167F, S194L |
| 159 | AC-NIHRD-PME4523 | Aceh | NSP3 | A58T, L897X, A898X, Y899X, C900X, N901X, K902X, T903X, V904X, G905X, L1244F, L1523X, G1524X, L1525X, A1526X, A1527X, I1528X, M1529X, Q1530X, L1531X, F1532X, F1533X |
|  |  |  | NSP6 | M52X, S53X, A54X, F55X |
|  |  |  | NSP12 | P323L, R349X, E350X, L351X, G352X, V353X, V354X, H355X, N356X, Q357X, D358X |
|  |  |  | Spike | D614G, K854X, F855X, N856X, G857X, L858X |
|  |  |  | NS3 | Q57H |
|  |  |  | NS8 | V62L |
| 160 | JT-NIHRD-PME3110 | Central Java | NSP3 3 | S284X, P822L, M914T |
|  |  |  | NSP12 | P323L |
|  |  |  | Spike | D614G, V1177L |
|  |  |  | NS3 | Q57H |
| 161 | KI-NIHRD-PME5182 | East Kalimantan | NSP13 | P47L |
|  |  |  | Spike | D614G |
|  |  |  | NS3 | Q57H |
| 162 | KT-NIHRD-PME4206-2 | Central Kalimantan | NSP4 | S395I |
|  |  |  | NSP6 | N40Y |
|  |  |  | NSP12 | P323L |
|  |  |  | NSP13 | V209I |
|  |  |  | Spike | D614G |
|  |  |  | NS3 | Q57H |
| 163 | KR-NIHRD-PME9874 | Riau Islands | NSP3 | A58T, K1211N, L1244F |
|  |  |  | NSP6 | D112N |
|  |  |  | NSP12 1 | P323L |
|  |  |  | NSP13 1 | V221F |
|  |  |  | Spike 2 | D614G, G744S |
|  |  |  | NS3 1 | Q57H |
|  |  |  | NS8 1 | V62L |
|  |  |  | N 1 | G238C |
| 164 | MU-NIHRD-PME8242 | North Maluku | NSP2 | Y242H, K557X |
|  |  |  | NSP3 | S609X, K610X, T611X, P822L, S1424X, L1425X, I1426X, Y1427X, S1428X, T1429X, A1430X, A1431X, L1432X, G1433X, V1434X, L1435X, M1436X, S1437X, N1438X, L1439X, G1440X, M1441X, P1442X |
|  |  |  | NSP4 | L329H, I345T, F351X, Y352X, L353X, T354X, N355X, D356X, V357X, S358X, F359X, L360X, A361X, H362X, I363X, Q364X, W365X, M366X, V367X, M368X, F369X, T370X, P371X, L372X, V373X, P374X, F375X, W376X, I377X, T378X |
|  |  |  | NSP12 | G44X, F45X, A46X, K47X, F48X, L49X, K50X, T51X, N52X, C53X, P323L |
|  |  |  | NSP13 | L526F |
|  |  |  | Spike | D614G, V911X, T912X, Q913X, N914X, V915X, L916X, Y917X, E918X, N919X, Q920X, K921X |
|  |  |  | NS3 | L41F, Q57H |
|  |  |  | N | S187L |
| 165 | JT-NIHRD-PME3225 | Central Java | NSP3 | P822L, F1354C |
|  |  |  | NSP12 | P323L |
|  |  |  | Spike | D614G, K811I |
|  |  |  | NS3 1 | Q57H |
|  |  |  | NSP3 | P822L, F1354C |
| 166 | KU-NIHRD-PME9235 | North Kalimantan | NSP8 | E23D |
|  |  |  | NSP12 | P323L |
|  |  |  | NSP13 | V226L |
|  |  |  | Spike | D614G |
|  |  |  | NS3 | Q57H |
|  |  |  | N | P199L |
